# Stress in Free-Roaming and Shelter-Housed Dogs: A Review of Neurobiological, Physiological and Behavioral Mechanisms Relevant to Welfare Assessment

**DOI:** 10.3390/ani16142183

**Published:** 2026-07-14

**Authors:** Gheorghița Rotaru, Teodor Daniel Hrițcu, Răzvan Nicolae Mălăncuș, Luminița Diana Hrițcu, Camelia Soponaru, Florin Nechifor, Vasile Boghian, Alexandra Andreea Cherșunaru, Alexandru Munteanu, Mihaela Claudia Spataru

**Affiliations:** 1 Faculty of Veterinary Medicine, University of Life Sciences “Ion Ionescu de la Brad”, Mihail Sadoveanu 8 Street, No. 3, 700490 Iasi, Romania; gheorghita.rotaru@iuls.ro (G.R.); teodor.hritcu@iuls.ro (T.D.H.); florin.nechifor@iuls.ro (F.N.); vasile.boghian@iuls.ro (V.B.); alexandra.chersunaru@iuls.ro (A.A.C.); alexandru.munteanu@iuls.ro (A.M.); mihaela.spataru@iuls.ro (M.C.S.); 2 Faculty of Psychology and Educational Sciences, Alexandru Ioan Cuza University, 700554 Iaşi, Romania; camelia.soponaru@uaic.ro

**Keywords:** free-roaming dogs, canine stress response, HPA axis, shelter-housed dogs cortisol biomarkers, behavioral indicators, animal welfare assessment, neuroendocrine stress

## Abstract

Stress is a major influencing behavior, health, and welfare in free-roaming dogs, particularly in environments characterized by unpredictability, social instability, and frequent human interactions. Although research on canine stress responses has expanded considerably, current knowledge remains fragmented across the neurobiological, physiological, and behavioral domains, limiting the development of comprehensive welfare assessment strategies. Importantly, free-roaming and shelter-housed dogs should not be viewed as entirely separate populations, since many shelter dogs originate from free-roaming environments and share similar stress-related experiences. This narrative review provides a comprehensive synthesis of current evidence on stress mechanisms in free-roaming and shelter-housed dogs The literature was explored across major scientific databases, and a total of 69 relevant studies were included. Particular emphasis was placed on the activation and regulation of the hypothalamic–pituitary–adrenal (HPA) axis, associated neuroendocrine alterations, and their behavioral expression. The strengths and limitations of commonly used physiological indicators, including cortisol measured in blood, saliva, feces and hair, were critically examined in relation to their applicability in real-world contexts. Behavioral manifestations of stress, such as fear- and anxiety-related responses, aggression, withdrawal, and stereotypic behaviors, were analyzed as functional outcomes of neurobiological activation and environmental challenges. Environmental stressors typical of shelter conditions as well as the role of human–dog interactions in stress modulation are also discussed. By combining neurobiological, physiological, and behavioral perspectives, this review highlights the importance of multidimensional stress assessment and proposes a conceptual framework linking biological mechanisms with observable behavioral indicators of welfare across the continuum from free-roaming to shelter environments. These findings may support the development of evidence-based welfare assessment and management strategies for free-roaming and shelter-housed dog populations.

## Simple Summary

Free-roaming dogs live in environments that are often unpredictable and stressful. These animals may experience food scarcity, conflicts with other dogs, exposure to harsh weather conditions, and frequent interactions with humans. Such challenges can affect their health, behavior, and overall well-being. Understanding how dogs respond to stress is essential for improving welfare and developing better management strategies in shelters and urban environments. This review examines how stress affects dogs by linking biological processes in the body with observable behavior. It discusses physiological responses to stressful situations, the use of biological indicators such as hormones to assess stress, and behavioral manifestations including fear, aggression, and withdrawal. The review also explores how environmental conditions and human interactions influence stress levels in dogs. By integrating findings from different scientific fields, this work highlights the importance of evaluating stress using multiple complementary indicators rather than relying on a single measure. These insights may help veterinarians, animal welfare specialists, and shelter staff better recognize signs of stress and implement strategies that improve the welfare of free-roaming and shelter-housed dogs.

## 1. Introduction

Stress is widely recognized as a major determinant of behavior, health, and welfare in dogs, arising from the interaction between environmental challenges, previous experiences, and individual biological mechanisms. In free-roaming dogs, repeated exposure to unpredictable living conditions, limited resources, social conflicts, and frequent interactions with urban environments represents a significant source of physiological and behavioral challenge [1,2,3,4].

Stress is commonly defined as the physiological and behavioral response of an organism to challenges that threaten homeostasis, as originally conceptualized by [5] and further refined in animal welfare science by [6]. In parallel, aggression represents a context-dependent behavioral strategy influenced by environmental pressures, individual state, and social interactions [7,8], and should be interpreted within a functional rather than purely pathological framework [9,10,11].

Free-roaming dogs are generally defined as dogs that live partially or entirely without direct human confinement or control, including stray, feral, and community-owned animals that depend to varying degrees on human-related resources. This population is highly heterogeneous, encompassing individuals with diverse life histories, ranging from previously owned dogs to animals born and raised in free-ranging environments.

The ecological and behavioral diversity of free-roaming dogs has been further characterized in the literature through classifications distinguishing between feral, stray, and community-owned dogs, each associated with different levels of human dependence and social organization [12,13,14,15,16]. These distinctions are important for understanding variability in stress exposure, social dynamics, and adaptive strategies across free-ranging populations.

Importantly, free-roaming dogs and shelter-housed dogs should not be considered as strictly separate populations, but rather as part of a dynamic continuum. A substantial proportion of dogs admitted to shelters originate from free-roaming environments, carrying with them prior experiences of environmental unpredictability, resource limitation, and variable human interaction. Consequently, stress responses observed in shelter settings may reflect both current environmental conditions and pre-existing adaptive or maladaptive mechanisms developed under free-roaming conditions.

This heterogeneity is reflected in the diversity of life histories, including abandoned animals, dogs originating from unstable domestic environments, and individuals born and raised outside direct human care. Such diversity in life history is not merely descriptive but constitutes an important determinant of variability in stress reactivity, coping strategies, and behavioral outcomes. As a result, the assessment of welfare in this population is particularly complex and methodologically challenging [17,18,19]. In addition, the transfer of free-roaming dogs to shelters, although often implemented for population management and public safety, represents an abrupt environmental transition that may intensify stress exposure and trigger significant behavioral changes [20,21]. In Romania, the population of free-roaming dogs has been estimated at approximately three million animals, particularly in urban environments where abandonment and uncontrolled reproduction contribute to long-term exposure to environmental stressors [22].

The physiological response to stress involves complex neurobiological mechanisms, primarily mediated by activation of the hypothalamic–pituitary–adrenal axis and the subsequent release of glucocorticoids, particularly cortisol. Experimental and observational studies have demonstrated that both acute and chronic exposure to stressors can induce measurable alterations in neuroendocrine functioning, with important consequences for behavior, immune competence, and overall health in dogs [20,23].

Under acute conditions, activation of the hypothalamic–pituitary–adrenal axis supports adaptive responses by mobilizing energy resources and increasing behavioral vigilance. However, when stressors are repeated or prolonged, sustained activation may result in the dysregulation of cortisol secretion and altered glucocorticoid receptor sensitivity, thereby influencing behavioral expression and coping capacity [24]. In shelter dogs, prolonged neuroendocrine activation has been associated with fear- and anxiety-related behaviors, aggression, and behavioral inhibition, suggesting that neurobiological dysregulation may directly translate into observable welfare impairment [24].

Behavioral manifestations of stress represent one of the most accessible tools for assessing the welfare of free-roaming dogs [25]. Behaviors such as excessive vocalization, avoidance of contact, stereotypies, or marked postural changes are frequently interpreted as indicators of compromised emotional state and may negatively influence adoption outcomes and management decisions [26,27,28]. Nevertheless, behavioral expression is highly context-dependent and shaped by individual temperament, life history, and environmental conditions, requiring cautious and standardized interpretation.

For this reason, stress assessment increasingly relies on the integration of behavioral indicators with physiological measurements, including cortisol determination in blood, saliva, feces, or hair. Although these biomarkers provide objective information on the activation of stress-response systems, their interpretation is influenced by individual variability, circadian rhythms, methodological differences, and in some cases, the stress induced by the sampling procedure itself [29,30]. Consequently, neither behavioral nor physiological indicators alone can fully capture the multidimensional nature of stress, highlighting the need for integrative welfare assessment approaches.

Over recent decades, increasing attention has been directed toward the role of shelter environments and human–dog interactions in modulating stress responses in free-roaming dogs. Welfare-oriented management strategies, including environmental enrichment, predictable routines, and positive human interactions, have been shown to reduce stress-related behavioral alterations and improve welfare outcomes [31,32]. These findings underline the importance of translating scientific knowledge into practical management frameworks.

Given the complexity of the biological mechanisms involved and the methodological diversity of stress assessment approaches, a comprehensive integrative perspective is required. This narrative review aims to synthesize current knowledge on the neurobiological, physiological, and behavioral mechanisms of stress in free-roaming and shelter-housed dogs, considered within a shared biological and ecological continuum. This perspective provides the conceptual basis for the present review and supports the integration of evidence from both free-roaming and shelter-housed dog populations. By integrating evidence from these complementary domains, this review proposes a conceptual framework linking neurobiological activation, physiological responses, and behavioral manifestations of stress with practical welfare assessment strategies. In addition, the review highlights current methodological limitations and knowledge gaps, particularly regarding the translation of experimental and shelter-based findings to real-world free-roaming contexts, thereby supporting the development of evidence-based management and welfare interventions.

## 2. Literature Search Strategy

The present study was designed as a structured narrative review aimed at integrating current knowledge on stress mechanisms in free-roaming and shelter-housed dogs. The literature search was conducted using major international scientific databases, including PubMed, Scopus, and Web of Science.

**Figure 1:**
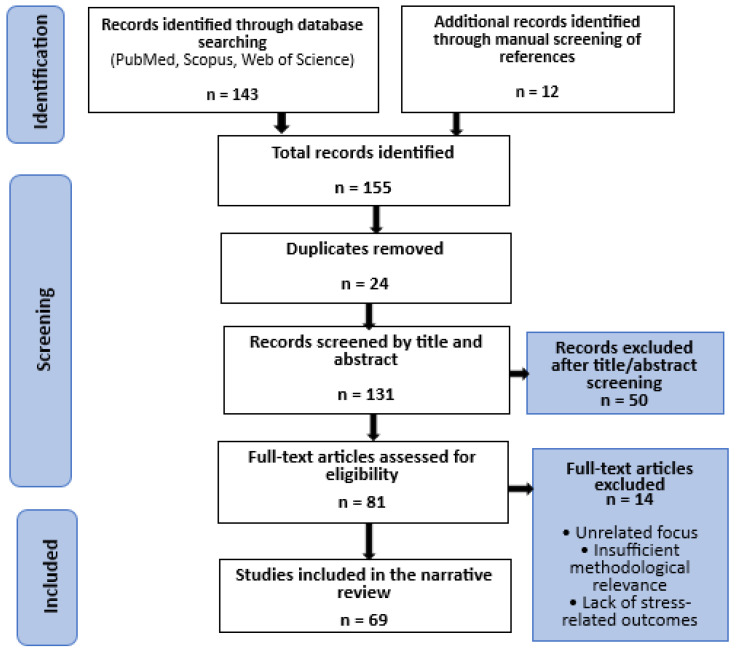
PRISMA-inspired flow diagram illustrating the literature selection process used in this narrative review. The literature selection process followed structured screening principles inspired by PRISMA recommendations adapted for narrative reviews.

The search strategy combined relevant keywords such as “free-roaming dogs”, “stray dogs”, “canine stress”, “animal welfare”, “HPA axis”, “cortisol”, “shelter environment” and “human–dog interaction”. Searches were primarily limited to articles published in English between 2000 and 2025. Earlier foundational publications were included selectively when considered essential for contextualizing core neurobiological, behavioral, and welfare-related concepts. The literature selection process followed structured screening principles inspired by the PRISMA 2020 Statement, adapted for narrative reviews. A flow diagram illustrating the literature selection process is presented in Figure 1.

The database search identified 143 records. An additional 12 records were identified through manual searching, resulting in a total of 155 records. After duplicate removal, 131 records were screened based on titles and abstracts. Subsequently, 81 full-text articles were assessed for eligibility. Following comprehensive evaluation of their methodological quality and relevance to the objectives of this narrative review, 69 studies met the predefined inclusion criteria and were included in the final qualitative synthesis (Figure 1).

Inclusion criteria comprised peer-reviewed studies addressing at least one of the following domains: neurobiological mechanisms of stress, physiological indicators (particularly cortisol and related biomarkers), behavioral manifestations of stress, and environmental or human-related factors influencing stress responses in dogs. Both experimental and observational studies were considered, including research conducted in free-roaming, shelter-housed, and companion dog populations.

Exclusion criteria included studies not directly related to stress mechanisms, articles focusing exclusively on unrelated clinical conditions without behavioral or physiological relevance, and publications lacking sufficient methodological clarity. Review articles were included selectively to support conceptual integration.

Given the biological continuity and frequent transition between free-roaming and shelter-housed dogs, studies conducted in shelter environments were included when considered relevant for understanding stress mechanisms applicable to dogs originating from free-roaming conditions.

The aim of this review was not to perform a systematic quantitative synthesis of the literature, but rather to provide a structured and integrative overview of current knowledge on stress mechanisms in dogs. By integrating evidence from different research domains, this review highlights the relationships between neurobiological activation, physiological responses, behavioral manifestations of stress, and welfare assessment. Although this narrative approach allows the integration of heterogeneous evidence, potential limitations related to selection bias, variability in study design, and the predominance of shelter-based research within the available literature are acknowledged. A limited number of self-citations were retained only when directly relevant to the thematic scope and conceptual framework of the review.

## 3. Results and Discussion

The literature included in this review comprised a total of 69 references, selected based on relevance to the neurobiological, physiological, and behavioral mechanisms of stress in dogs. The evidence base included a combination of experimental studies, observational research and review articles.

A substantial proportion of the available studies were conducted in controlled or semi-controlled environments, particularly in shelter-housed or companion dog populations, reflecting the current structure of scientific literature in this field. Fewer studies specifically addressed free-roaming dog populations in naturalistic or urban environments, highlighting a relative gap in ecologically oriented research.

The included studies investigated a range of domains, including activation of the hypothalamic–pituitary–adrenal axis, physiological stress indicators (particularly cortisol), behavioral manifestations of stress, and environmental and human-related factors influencing stress responses. This diversity of approaches supports an integrative analysis while also requiring careful interpretation in relation to differences in study design and environmental context.

### 3.1. Neurobiological Mechanisms of Stress in Dogs

Stress in dogs involves the coordinated activation of multiple neurobiological systems that regulate adaptive responses to environmental challenges. Rather than representing a single linear pathway, the stress response reflects dynamic interactions between endocrine, autonomic, and central nervous system processes. Central to this response is the activation of the hypothalamic–pituitary–adrenal (HPA) axis, which integrates the perception of stressors with endocrine and behavioral outputs [23,33].

At the hypothalamic level, exposure to a stressor triggers the release of corticotropin-releasing hormone (CRH), stimulating adrenocorticotropic hormone (ACTH) secretion from the anterior pituitary and the subsequent release of glucocorticoids—primarily cortisol—from the adrenal cortex. Although frequently described as a sequential endocrine cascade, the functional relevance of this pathway lies in its capacity to modulate energy mobilization, immune activity and behavioral vigilance [34]. Under acute stress conditions, activation of the HPA axis facilitates adaptive responses by enabling rapid physiological and behavioral adjustments to environmental demands [35].

However, when exposure to stressors becomes repeated or prolonged, sustained HPA axis activation may lead to alterations in cortisol secretion patterns and changes in glucocorticoid receptor sensitivity, reflecting processes of neuroendocrine dysregulation rather than adaptive regulation [35,36]. In dogs, chronic stress has been associated with either elevated basal cortisol concentrations or blunted HPA responses, depending on the duration and intensity of exposure as well as individual variability [20,37,38]. Moreover, prolonged neuroendocrine dysregulation may increase susceptibility to stress-associated pathological changes, highlighting the complexity of chronic stress responses and reinforcing the need for integrative approaches to stress assessment [35,36]. Such findings highlight the complexity of interpreting cortisol values in isolation and reinforce the need for integrative approaches to stress assessment.

In addition to the HPA axis, the autonomic nervous system plays a crucial role in mediating immediate stress responses. Activation of the sympathetic branch and the release of catecholamines, including adrenaline and noradrenaline, contribute to rapid physiological adjustments such as increased heart rate, heightened arousal, and behavioral vigilance [2]. These autonomic responses interact with endocrine mechanisms to shape the intensity and expression of behavioral reactions to stress.

This autonomic response is mediated through the sympathetic–adreno-medullary system and plays a critical role in acute stress adaptation, particularly in unpredictable environments such as those encountered by free-roaming dogs.

At the central nervous system level, brain structures including the amygdala, hippocampus, and prefrontal cortex are critically involved in the appraisal and regulation of stress-related stimuli. The amygdala contributes to threat detection and the initiation of fear responses, while the hippocampus participates in contextual memory and negative feedback regulation of the HPA axis [35,39]. Chronic stress may induce structural and functional alterations within these regions, potentially impairing emotional regulation, behavioral flexibility, and coping capacity.

Canine-specific studies have similarly demonstrated that chronic exposure to stressors such as prolonged shelter confinement, social instability, and unpredictable human interaction may alter neuroendocrine regulation and behavioral responsiveness in dogs. Increased cortisol reactivity, altered coping patterns, and stress-associated behavioral inhibition have been documented particularly in shelter-housed populations, supporting the applicability of these neurobiological mechanisms within canine-specific contexts [20,24,31].

In free-roaming and shelter-housed dogs, repeated exposure to unpredictable environmental conditions, spatial restriction, noise, and inconsistent human interactions may sustain neuroendocrine activation and compromise adaptive regulation. Empirical studies indicate that shelter environments can amplify HPA axis activation and are associated with fear- and anxiety-related behaviors, defensive aggression, and behavioral inhibition [20,24]. These observations suggest that neurobiological dysregulation may directly contribute to observable behavioral alterations and impaired welfare.

### 3.2. Physiological Indicators of Stress in Dogs

Physiological indicators are widely employed to quantify stress responses in dogs, providing objective measures of neuroendocrine and autonomic activation. However, their interpretative value depends not only on analytical precision, but also on contextual factors, individual variability, and the temporal dynamics of stress exposure [29,33]. In free-roaming and shelter-housed dogs, these variables become particularly relevant, as environmental instability, unpredictable stimuli, and heterogeneous life histories may substantially influence baseline physiological values and stress reactivity patterns.

#### 3.2.1. Cortisol as the Primary Marker of Stress

Cortisol remains the most extensively investigated biomarker of stress in dogs and is commonly used to evaluate activation of the hypothalamic–pituitary–adrenal (HPA) axis [20,23]. Despite its widespread application, interpretation of cortisol concentrations requires careful consideration of the sampling matrix, timing of collection, environmental context, and individual baseline variability.

Substantial inter-individual differences in basal cortisol levels have been documented even in clinically healthy dogs, highlighting the limitations of relying on single measurements as universal indicators of stress (Table 1).

The findings summarized in Table 1 clearly illustrate that basal cortisol concentrations in dogs exhibit considerable inter-individual variability, even under clinically normal conditions. This variability is influenced by factors such as breed, age, temperament, prior experiences and environmental context, making it difficult to establish a universal reference value applicable to all individuals.

Importantly, repeated measurements within the same individual often demonstrate greater consistency than comparisons across populations, suggesting that individual baseline profiling may provide a more reliable framework for interpreting stress responses. This consideration is particularly relevant in free-roaming and shelter-housed dogs, where heterogeneous life histories and fluctuating environmental conditions may substantially affect the baseline endocrine parameters.

Consequently, isolated cortisol measurements should be interpreted with caution and ideally combined with longitudinal assessment and behavioral evaluation. Without such integration, there is a risk of either overestimating or underestimating stress status based solely on endocrine data.

Cortisol can be measured using various biological matrices, each characterized by specific methodological advantages and interpretative limitations. Plasma cortisol provides precise information on endocrine activation; however, blood sampling is inherently invasive and may itself trigger an additional stress response, potentially confounding the obtained values [29]. For this reason, its applicability in applied studies involving free-roaming dogs is often limited.

Salivary cortisol represents a less invasive alternative and is widely employed in the assessment of acute stress responses [43]. Because salivary concentrations reflect the free, biologically active fraction of cortisol, they are particularly suitable for detecting rapid endocrine fluctuations. Nevertheless, results may be influenced by sampling time, circadian variation, handling procedures, or sample contamination, factors that may introduce variability in field conditions [23,30].

In the context of acute stress, cortisol measurements have proven useful for identifying rapid endocrine activation in response to short-term environmental challenges. Transient elevations in cortisol concentrations have been documented following stressors such as transport, veterinary examination, hospitalization, or surgical procedures, as summarized in Table 2.

Importantly, such acute increases are generally considered adaptive, reflecting the organism’s capacity to mobilize physiological resources in response to immediate demands. In contrast to chronic stress, where dysregulation may occur, acute cortisol elevations do not necessarily indicate compromised welfare, but rather functional activation of stress-response systems.

In the assessment of chronic stress, fecal and hair cortisol measurements have gained increasing attention as indicators of prolonged endocrine activation [46,47]. Unlike plasma or salivary cortisol, which primarily reflect short-term fluctuations, fecal cortisol metabolites provide an integrated measure of glucocorticoid secretion over an extended period, thereby reducing the influence of transient acute stress responses. However, interpretation remains dependent on gastrointestinal transit time, metabolic processing, and individual variability [46,48].

Hair cortisol analysis offers an additional tool for evaluating long-term stress exposure, as cortisol is incorporated into the hair shaft during growth. This method has been considered particularly relevant for assessing welfare in shelter-housed dogs or animals subjected to sustained environmental stressors [4,30,49,50]. Nevertheless, factors such as coat characteristics, hair growth rate, and potential external contamination may influence the measured concentrations.

In addition to cortisol, other emerging biomarkers have been investigated as indicators of stress and physiological status. These include salivary immunoglobulins, which reflect immune function, and gut microbiome composition, which may be influenced by chronic stress and environmental conditions. Such parameters offer promising perspectives for the non-invasive assessment of stress and health, particularly in free-roaming dog populations where repeated sampling may be challenging.

Under conditions of chronic or prolonged stress, the relationship between cortisol levels and stress status becomes increasingly complex. Rather than displaying consistently elevated concentrations, dogs exposed to sustained stress may exhibit cumulative increases, attenuated responses, or inconsistent hypothalamic–pituitary–adrenal axis reactivity, reflecting processes of neuroendocrine adaptation or dysregulation, as summarized in Table 3.

The patterns summarized in Table 3 highlight the non-linear relationship between chronic stress exposure and cortisol dynamics in dogs. Unlike acute stress responses, which are typically characterized by transient elevations, chronic stress may lead to heterogeneous endocrine profiles, including sustained increases, attenuated responses, or fluctuating reactivity. Such variability reflects complex processes of neuroendocrine adaptation, and in some cases, dysregulation of the hypothalamic–pituitary–adrenal axis.

These findings underscore an important methodological consideration: elevated cortisol levels do not necessarily indicate greater welfare impairment, and normal or reduced values do not always reflect the absence of stress. In chronically stressed dogs, particularly those housed long-term in shelters or exposed to unstable environments, blunted endocrine responses may represent physiological exhaustion or altered feedback sensitivity rather than resilience. Consequently, the interpretation of chronic cortisol patterns should be integrated with behavioral observations and environmental context in order to avoid the misclassification of stress status.

In addition to cortisol-based measures, autonomic indicators such as heart rate (HR) and heart rate variability (HRV) are increasingly used to evaluate stress responses in dogs. HRV, in particular, provides valuable information regarding autonomic balance and adaptive capacity, with reduced variability frequently associated with heightened stress and impaired emotional regulation. These measures may complement endocrine indicators by providing the real-time assessment of autonomic activation under both acute and chronic stress conditions. Moreover, combining endocrine and autonomic parameters may improve the integrated interpretation of stress responses in free-roaming and shelter-housed dogs [29,33,43,51].

#### 3.2.2. Other Physiological Indicators of Stress

In addition to cortisol-based measurements, several other physiological parameters have been investigated as potential indicators of stress in dogs. Cardiovascular variables, particularly heart rate and heart rate variability (HRV), reflect autonomic nervous system activity and provide insight into the balance between sympathetic and parasympathetic regulation. Reduced heart rate variability has been associated with heightened stress and anxiety states, suggesting altered autonomic regulation under challenging conditions [51,52]. However, accurate HRV assessment requires standardized measurement protocols and specialized equipment, which may limit its applicability in free-roaming or shelter environments.

Immunological indicators, including changes in leukocyte profiles or neutrophil-to-lymphocyte ratios, have also been proposed as indirect markers of stress-related physiological activation [33]. While such parameters may reflect chronic stress exposure, they are influenced by numerous physiological, environmental, and pathological factors, which reduces their specificity as standalone indicators of stress.

Taken together, these findings suggest that although alternative physiological markers may complement cortisol assessment, none can provide a comprehensive assessment of stress when considered in isolation. Their interpretative value increases when integrated with behavioral indicators and environmental context, reinforcing the integrative nature of stress evaluation in free-roaming and shelter-housed dogs.

#### 3.2.3. Limitations and the Need for an Integrative Approach

Although physiological indicators provide valuable objective data, their isolated use may lead to incomplete or potentially misleading conclusions. Individual variability, environmental influences, and methodological differences among studies represent major challenges when comparing results. Particularly in free-roaming dogs, living conditions and individual life history may significantly influence physiological stress responses [31].

Therefore, the scientific literature emphasizes the importance of integrating physiological indicators with behavioral assessments for a more accurate evaluation of stress levels and animal welfare. Such an integrative approach allows for a more nuanced interpretation of stress responses and supports effective management strategies aimed at improving the welfare of free-roaming and shelter-housed dogs [42,53,54].

### 3.3. Behavioral Manifestations of Stress in Dogs

Behavior represents one of the most accessible and ecologically valid indicators of stress in dogs, providing direct insight into emotional state and coping capacity within a given environmental context [24,26]. Unlike isolated physiological measurements, behavioral expression reflects the functional outcome of neurobiological activation and interactions with environmental conditions.

Stress-related behaviors in free-roaming and shelter-housed dogs are not uniform but vary according to stressor intensity, duration, and individual life history. These manifestations can broadly be categorized into fear- and anxiety-related behaviors, aggressive responses, withdrawal and apathy, as well as repetitive or stereotypic behaviors [2]. It is important to distinguish between different forms of repetitive behaviors, such as stereotypies and compulsive disorders (e.g., acral licking), which may have distinct motivational and neurobiological underpinnings. While stereotypies are often associated with environmental restriction and frustration, compulsive behaviors may be more closely related to individual predisposition and altered neural processing. These patterns may also be influenced by personality traits, further contributing to variability in behavioral responses.

Importantly, these categories do not merely describe observable patterns but reflect distinct coping strategies and adaptive or maladaptive responses to environmental challenges.

The use of standardized ethograms provides a structured framework for the identification and interpretation of stress-related behaviors. Behavioral classification systems, such as those described by [55], provide practical guidance for recognizing postural changes, facial expressions and activity patterns associated with stress in dogs, thereby improving the consistency and reliability of behavioral assessments.

Individual differences in temperament, often described along a shy–bold continuum, have been shown to significantly influence stress responsiveness and coping strategies in animals. In free-roaming dogs, personality traits may modulate behavioral expression, social interactions, and adaptation to environmental challenges, contributing to variability in observed stress indicators.

In addition to direct behavioral observation and ethogram-based approaches, standardized assessment tools such as the Canine Behavioral Assessment and Research Questionnaire (C-BARQ) and Qualitative Behavioral Assessment (QBA) have been increasingly applied in canine welfare research. These instruments allow for the structured evaluation of behavioral tendencies, emotional expression, and stress-related responses, including fearfulness, aggression, sociability, and coping patterns. In shelter environments, behavioral assessment protocols may also assist in identifying dogs at risk of chronic stress, reduced adaptability, or impaired welfare [56,57].

Such behavioral manifestations are widely recognized as key indicators of stress; however, their welfare implications differ depending on context and duration of exposure. While some patterns may represent adaptive responses to acute challenges, others are associated with chronic stress, reduced coping capacity, and potential welfare compromise. Distinguishing between transient stress reactions and persistent behavioral alterations is therefore essential when interpreting behavioral data in free-roaming and shelter-housed dogs.

The principal behavioral categories and their welfare implications are presented in Table 4.

#### 3.3.1. Fear- and Anxiety-Related Behaviors

Fear and anxiety represent some of the most frequently observed behavioral manifestations of stress in dogs. Although often used interchangeably in applied contexts, fear typically refers to a response to an immediate and identifiable threat, whereas anxiety is characterized by anticipatory apprehension in the absence of a clearly defined stimulus. This distinction is particularly relevant for welfare assessment, as chronic anxiety may persist beyond the original stressor and affect broader behavioral functioning.

Behavioral expressions of fear and anxiety include trembling, lowered body posture, tail tucked between the legs, avoidance of eye contact, excessive vocalization, and escape attempts. These responses are generally associated with the perception of threat and activation of neurobiological stress pathways, particularly those involving the hypothalamic–pituitary–adrenal axis and limbic system structures. In shelter environments, dogs frequently display heightened fear responses to unfamiliar noises, the presence of conspecifics, or handling by unfamiliar individuals [20,37], reflecting the impact of unpredictable and potentially aversive environmental conditions.

In addition, separation-related behaviors have been associated with increased reactivity to everyday situations that may elicit frustration or fear, suggesting processes of emotional generalization beyond the initial context [58]. Such generalization may contribute to persistent hypervigilance and exaggerated stress-related behavioral responses, particularly in dogs exposed to repeated or uncontrollable stressors.

From a functional perspective, fear- and anxiety-related behaviors may initially represent adaptive responses aimed at avoiding or mitigating perceived threats. However, when these behaviors become persistent, exaggerated, or contextually inappropriate, they may indicate impaired coping capacity and reduced adaptability to environmental challenges. In free-roaming and shelter-housed dogs, chronic expression of fear-related behaviors is often associated with prolonged stress exposure and may contribute to emotional dysregulation and behavioral instability.

Chronic anxiety can further impair adaptive capacity by reducing behavioral flexibility and compromising the formation of stable human–dog relationships. These patterns are associated with decreased adoption prospects and an increased risk of long-term behavioral problems [27]. Evidence from experimental animal models further supports the link between prolonged stress exposure and anxiety-related behavioral alterations, highlighting shared neurobiological pathways underlying stress-induced emotional dysregulation across species [59].

#### 3.3.2. Aggressive Behaviors

Aggressive responses may emerge as behavioral manifestations of stress, particularly when dogs are confronted with situations perceived as threatening and lack effective coping alternatives. In such contexts, aggression can represent a defensive strategy aimed at increasing distance from the stressor rather than an expression of stable temperament traits.

From a neurobiological perspective, stress-related aggression is often associated with heightened arousal and activation of stress-response systems, including the hypothalamic–pituitary–adrenal axis and limbic structures involved in threat processing. These mechanisms may lower behavioral thresholds for reactive behaviors and reduce the capacity for flexible behavioral regulation.

In shelter environments, defensive aggression directed toward humans or conspecifics has been frequently reported, where spatial restriction, competition for resources, and repeated handling may intensify stress levels [2,24]. Under these conditions, aggression may reflect increased arousal and reduced behavioral flexibility rather than an inherent predisposition. Similar patterns may also be observed in free-roaming dogs exposed to unpredictable or aversive human interactions.

From a functional perspective, aggressive behavior may initially serve an adaptive role by enabling the animal to cope with perceived threats. However, when such responses become persistent or exaggerated, they may indicate impaired coping capacity and sustained emotional dysregulation. It is therefore essential to distinguish stress-induced aggression from trait-based or learned forms of aggression, as misinterpretation of context-dependent aggressive responses may lead to inappropriate management decisions or reduced adoption opportunities.

From a welfare perspective, persistent stress-related aggression may reflect chronic stress exposure and compromised adaptive capacity, requiring targeted environmental and behavioral interventions aimed at reducing stress and improving behavioral stability.

#### 3.3.3. Avoidance Behaviors and Social Withdrawal

In contrast to overt aggression, some dogs respond to stress through avoidance behaviors and social withdrawal, reflecting a more passive coping strategy. Such behaviors may include immobility, hiding, reduced exploratory activity, and diminished social interaction with conspecifics and humans. Although less disruptive from a management perspective, these patterns may indicate significant emotional distress and reduced engagement with the environment.

In free-roaming and shelter-housed dogs, prolonged exposure to uncontrollable or unpredictable stressors may promote behavioral inhibition and reduced responsiveness to environmental stimuli. From a neurobiological perspective, such responses are consistent with the chronic activation and potential dysregulation of stress-response systems, which may favor behavioral suppression over active coping strategies. These manifestations have been interpreted as signs of learned helplessness or apathy, particularly in chronically stressed individuals [4,31].

From a functional perspective, avoidance behaviors may initially serve an adaptive role by reducing exposure to perceived threats. However, when these responses become persistent, they may indicate impaired coping capacity and diminished behavioral flexibility. In such cases, reduced exploration and social interaction may reflect impaired coping capacity rather than behavioral stability.

From a welfare standpoint, social withdrawal and behavioral suppression are strongly associated with compromised welfare and reduced adaptability. Dogs displaying persistent withdrawal are often perceived as less adoptable, which may prolong shelter stay and further reinforce stress-related behavioral patterns. Consequently, passive stress responses should be interpreted with the same level of concern as overt aggressive reactions.

#### 3.3.4. Repetitive and Stereotypic Behaviors

Repetitive and stereotypic behaviors, such as pacing, circling, excessive licking, or bar-biting, are widely recognized as behavioral indicators of chronic stress and environmental frustration in dogs [26,60]. Unlike goal-directed actions, stereotypies are characterized by repetitive, invariant patterns that appear to lack an obvious functional purpose.

These behaviors are commonly observed in kennel or shelter environments where spatial restriction, limited environmental stimulation, and reduced behavioral control may compromise adaptive coping. From a neurobiological perspective, the emergence of stereotypies has been associated with the sustained activation of stress-response systems and potential dysregulation of neural circuits involved in behavioral control and reward processing. In this context, stereotypic behaviors may reflect a coping attempt associated with prolonged stress exposure rather than meaningless activity.

From a functional perspective, repetitive behaviors may initially represent coping responses aimed at reducing internal arousal or environmental uncertainty. However, when they become persistent and inflexible, they are generally interpreted as indicators of chronic stress and reduced behavioral adaptability. In free-roaming and shelter-housed dogs, such patterns are often associated with prolonged environmental restriction and limited opportunities for species-specific behaviors.

From a welfare perspective, the presence of stereotypic behavior suggests persistent environmental inadequacy or frustration and may indicate that the animal’s coping capacity has been exceeded. Although such behaviors may decrease following environmental enrichment or improved management conditions, their occurrence should prompt the evaluation of housing conditions and implementation of targeted stress-reduction strategies.

#### 3.3.5. Limitations of Behavioral Assessment

Although behavioral observation is fundamental for evaluating stress in dogs, it is subject to important interpretative limitations. Behavioral expression is inherently context-dependent and may vary according to environmental conditions, timing of assessment, and the presence of observers. Furthermore, similar behaviors may have different functional meanings depending on the animal’s previous experiences and emotional state.

Observer bias and inconsistencies in behavioral scoring systems may further affect reliability, particularly in applied shelter settings. In addition, certain stress-related behaviors may be subtle or suppressed, leading to the potential underestimation of stress levels. Conversely, isolated behaviors interpreted as stress indicators may represent transient adaptive responses rather than sustained welfare compromise.

From a conceptual perspective, behavioral data alone cannot fully capture the internal physiological processes underlying stress responses. As highlighted in integrative frameworks of animal emotion [61], accurate interpretation requires the consideration of cognitive appraisal, affective state, and environmental context.

Moreover, the lack of standardized behavioral assessment protocols represents an additional challenge, particularly in heterogeneous populations such as free-roaming and shelter-housed dogs. Variability in assessment methods may limit comparability across studies and hinder the development of consistent welfare assessment tools.

Therefore, combining behavioral observations with physiological indicators and contextual information remains essential for a comprehensive and reliable assessment of stress in free-roaming and shelter-housed dogs. Such integrative approaches may support the development of more standardized and evidence-based welfare assessment frameworks in applied settings.

### 3.4. Stress in the Shelter Environment and Environment-Specific Factors Affecting Free-Roaming Dogs

Although a substantial proportion of the available literature on canine stress originates from studies conducted in shelter environments, these findings remain highly relevant for understanding stress mechanisms in free-roaming dogs. This is due to both the shared biological foundations of stress responses and the frequent transition of individuals from free-roaming conditions into shelter systems. However, it is important to recognize that free-roaming dogs are exposed to additional environmental challenges, including resource unpredictability, human–animal conflict, traffic, disease exposure, and climatic variability, which may further influence stress expression and coping strategies.

The shelter environment represents one of the most frequently studied contexts in which stress responses in dogs may be intensified and persist over time. Although shelters aim to protect free-roaming dogs and facilitate population management, conditions of confinement may act as major stressors affecting behavior and welfare [20,24].

One of the primary stressors in shelters is spatial restriction, which limits opportunities for exploration and the expression of species-typical behaviors. Confinement in individual kennels may promote frustration, stereotypic behaviors, and social withdrawal, particularly in dogs with high activity levels or a life history in open environments [26] A perceived lack of control over the environment and reduced predictability of daily events are considered central factors contributing to chronic stress and impaired coping.

Environmental noise represents another important stressor in shelters, generated by vocalizations of other dogs, routine staff activities, and the presence of visitors. High and unpredictable noise levels have been associated with increased cortisol concentrations and an intensification of fear- and anxiety-related behaviors [21,31]. Continuous noise exposure may impair rest and behavioral recovery, thereby increasing cumulative stress load.

Both social isolation and overcrowding may further exacerbate stress. Inadequate opportunities for stable social interactions—either with conspecifics or humans—may contribute to emotional deterioration and withdrawal, whereas overcrowding may promote competition for resources, conflict escalation, and an increased incidence of aggressive behaviors [24]. In this context, management practices that balance social needs with stress prevention are particularly relevant.

Frequent and often unpredictable handling by staff or volunteers represents an additional stressor, particularly for free-roaming dogs with previous negative experiences involving humans. Cleaning procedures, veterinary examinations, and repeated relocation between kennels may trigger recurrent acute stress responses and contribute to prolonged physiological activation [20,27]. Length of stay is also a critical factor, as prolonged shelter confinement is associated with increased risk of behavioral problems and reduced adoption prospects [31].

Taken together, these factors do not act in isolation but contribute cumulatively to the overall stress burden experienced by shelter-housed dogs. The interaction between multiple environmental stressors may lead to the sustained activation of stress-response systems and increased allostatic load, thereby reducing adaptive capacity and increasing vulnerability to behavioral and physiological dysregulation.

Although shelter-housed dogs experience multiple confinement-related stressors, free-roaming dogs are exposed to a different but equally complex set of environmental challenges. Unlike dogs living in shelters, free-roaming individuals cannot always avoid or retreat from stressful situations, such as territorial conflicts, traffic, human harassment, competition for limited resources, or adverse weather conditions. Instead, they must continuously adapt to dynamic and often unpredictable environments, requiring sustained behavioral flexibility and prolonged physiological activation. This ecological contrast resembles the distinction described in zoo animal welfare research between captive animals, whose stressors are largely associated with environmental restriction and limited behavioral control, and free-ranging wildlife, which faces greater environmental uncertainty but retains the opportunity to respond through movement and behavioral choice [62,63,64]. Consequently, the interpretation of stress in free-roaming dogs should consider not only the presence of environmental stressors, but also the animals’ capacity to cope with them through adaptive behavioral strategies.

In this context, the scientific literature emphasizes the importance of implementing evidence-based stress-reduction strategies in shelter management, including environmental enrichment, predictable routines, noise mitigation, structured positive social interactions, and staff training in the recognition of stress-related behaviors. Such interventions have been associated with measurable reductions in stress indicators and improvements in behavioral outcomes, ultimately contributing to enhanced welfare and increased adoption success in free-roaming dogs [26,32].

In free-roaming dogs, these stressors may be further compounded by additional environmental pressures not typically encountered in shelter settings, such as competition for food resources, exposure to urban hazards, and inconsistent or negative human interactions. Unlike shelter-housed dogs, free-roaming individuals often have greater opportunities to express natural behaviors and avoid certain stressors through movement; however, they are simultaneously exposed to unpredictable ecological challenges over which they have limited control. These considerations highlight the importance of interpreting stress responses within their ecological context when extrapolating shelter-based findings to free-roaming populations.

### 3.5. The Role of Human–Dog Interaction in Stress Modulation

The nature and impact of human–dog interaction may differ substantially between free-roaming and shelter-housed dogs, depending on prior experiences, degree of human dependence, and environmental context.

Human–dog interaction represents a central modulatory factor in canine stress responses, particularly in free-roaming and shelter-housed dogs. As a result of domestication and coevolution, dogs are highly sensitive to human social cues, emotional states, and behavioral consistency [19]. The quality and predictability of human interaction may therefore either attenuate or exacerbate physiological and behavioral stress responses.

For free-roaming dogs, prior experiences with humans vary widely, ranging from affiliative contact to traumatic exposure. These experiences shape cognitive appraisal of human presence and influence behavioral reactivity in shelter environments [2]. Inconsistent or aversive interactions may reinforce fear, defensive aggression, or withdrawal, whereas structured and positive contact may facilitate emotional regulation and adaptive coping.

From a physiological perspective, positive human interaction may act as a buffering factor against stress by modulating activation of the hypothalamic–pituitary–adrenal axis and reducing physiological arousal. Empirical studies indicate that calm tactile contact, consistent verbal communication, and structured play sessions are associated with reductions in physiological stress markers and improvements in behavioral expression in shelter dogs [21,31]. Conversely, coercive or unpredictable handling may intensify stress activation and contribute to behavioral deterioration [24]. From a welfare perspective, perceived lack of control and inconsistent signaling during human interaction represent critical stress-enhancing factors.

An additional dimension of human–dog interaction is emotional transfer or “emotional contagion”, whereby dogs may respond both behaviorally and physiologically to human emotional states [31,65,66]. Recent studies have further demonstrated that dogs are capable of detecting and synchronizing with human emotional cues, both at behavioral and physiological levels [67]. Evidence suggests that human stress can influence canine cortisol levels and behavioral responses, highlighting the bidirectional nature of the human–dog relationship. These findings emphasize the importance of consistency, predictability, and emotional regulation in human–dog interactions, particularly in shelter environments.

Human interaction also directly influences adoption outcomes. In this context, facial expressions also play an important role in human perception and adoption decisions. Studies using the Dog Facial Action Coding System (DogFACS) have shown that specific facial movements, such as the inner eyebrow raise [68], often referred to as “puppy eyes”, are associated with increased human attention and improved adoption success. Dogs displaying affiliative and socially responsive behaviors are generally perceived as more adoptable, whereas fear-based or stress-related behaviors may reduce adoption likelihood [27,32]. Accordingly, structured and evidence-based socialization programs may improve both welfare and adoption outcomes.

Taken together, these findings highlight that human–dog interaction is not merely a contextual factor but a central determinant of stress modulation, with important implications for physiological regulation, behavioral adaptation, and welfare outcomes in free-roaming and shelter-housed dogs.

#### Implications for Welfare Assessment and Management of Free-Roaming Dogs

**Figure 2:**
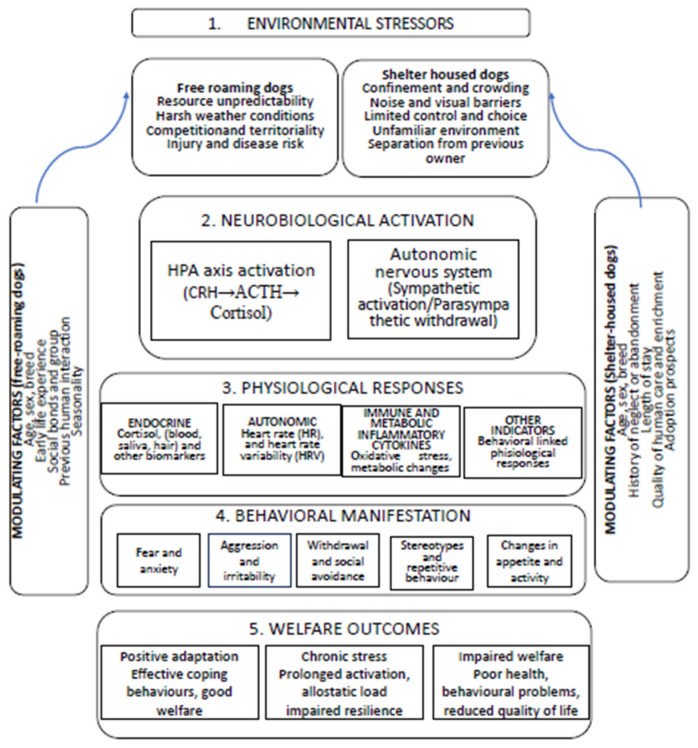
Conceptual integrative framework linking environmental stressors, neurobiological activation, physiological responses, behavioral manifestations, and welfare outcomes in free-roaming and shelter-housed dogs.

Based on the evidence synthesized throughout this review, stress in free-roaming and shelter-housed dogs may be conceptualized within an integrative framework linking environmental stressors, neurobiological activation, physiological responses, and behavioral manifestations. Within this framework, environmental conditions and prior life history modulate activation of the hypothalamic–pituitary–adrenal axis and autonomic pathways, which in turn influence endocrine responses, emotional regulation, and observable behavioral outcomes. The interaction between these domains ultimately shapes welfare status and coping capacity, highlighting the need for multidimensional and context-sensitive assessment strategies, as illustrated in Figure 2.

In the specific context of free-roaming dogs, welfare assessment presents additional challenges compared to controlled environments such as shelters. The absence of direct human supervision, variability in resource availability, and exposure to complex urban or natural environments require the use of flexible and context-adapted assessment strategies. Consequently, behavioral indicators and non-invasive physiological measures become particularly valuable tools for evaluating stress in these populations, especially when direct handling or repeated sampling is not feasible.

Understanding the neurobiological mechanisms of stress, associated physiological indicators, and behavioral manifestations has direct implications for welfare assessment and management strategies in free-roaming and shelter-housed dogs. Evidence consistently indicates that welfare cannot be reliably evaluated using a single parameter, but requires a multidimensional and context-sensitive approach combining behavioral observation with appropriate physiological measures [33,61].

Given the substantial individual variability in stress responsiveness—shaped by life history, previous human interactions, duration of exposure to stressors, and coping capacity—assessment protocols should prioritize longitudinal monitoring and individual profiling. The combined use of validated behavioral assessment tools and non-invasive physiological markers, such as salivary cortisol, may enhance accuracy in identifying dogs at risk of chronic stress [31].

From a management perspective, early identification of heightened stress states is essential to prevent behavioral deterioration and health compromise. Structured monitoring protocols, alongside targeted environmental and social interventions, may facilitate adaptation, support recovery, and reduce cumulative stress load [24].

Welfare-oriented strategies—including environmental optimization, predictable routines, enrichment, and consistent positive human interaction—have demonstrated measurable benefits in reducing stress-related behaviors and improving overall welfare [26,32]. In addition, staff training in stress recognition, low-arousal handling techniques, and awareness of emotional transfer processes plays a critical role in sustaining effective stress mitigation [65].

Integrating welfare assessment into adoption strategies represents a further practical implication. Dogs benefiting from evidence-based stress-reduction interventions tend to display more adaptive behavioral profiles and improved social responsiveness, which are positively associated with adoption outcomes [27,69].

Overall, integrating neurobiological, physiological, and behavioral data within an applied management framework provides a strong basis for evidence-based decision-making and the development of effective, ethically grounded strategies for stress reduction in free-roaming dog populations.

In this context, the interpretation of stress indicators should account for environmental variability and individual life history, which may influence both behavioral expression and physiological responses. This highlights the need for adaptive, field-oriented welfare assessment frameworks specifically tailored to free-roaming populations.

## 4. Conclusions

Stress in free-roaming and shelter-housed dogs represents a complex and multidimensional phenomenon arising from the interaction between neurobiological mechanisms, environmental stressors, and individual life history. The activation of the hypothalamic–pituitary–adrenal axis, autonomic responses, and associated behavioral manifestations together shape the observable expression of stress and its welfare implications.

The present narrative review highlights that neither physiological indicators nor behavioral observations alone are sufficient for accurate welfare assessment. Instead, an integrative framework combining neuroendocrine measures, validated behavioral evaluation, and contextual analysis provides a stronger basis for distinguishing between adaptive stress responses, chronic dysregulation, and compromised coping capacity.

Importantly, this review emphasizes that stress responses in free-roaming and shelter-housed dogs cannot be fully understood without considering the cumulative and context-dependent effects of environmental conditions. Shelter environments represent complex stress landscapes in which spatial restriction, unpredictability, social dynamics, and human interaction interact to modulate stress expression over time.

A key contribution of this review is the integrative perspective linking neurobiological, physiological, and behavioral dimensions of stress across free-roaming and shelter-housed dog populations. By synthesizing evidence from these interconnected domains within a unified conceptual framework applicable to real-world conditions, this review highlights the importance of multidimensional and context-sensitive approaches for understanding stress responses and welfare outcomes in dogs. By linking mechanistic understanding with observable indicators, this perspective may support more accurate welfare assessment and facilitate the development of targeted, evidence-based management strategies. However, an important limitation of the current literature is the relatively limited number of studies conducted in naturalistic free-roaming contexts compared to the predominance of research in shelter, laboratory, or clinical settings. This highlights the need for further ecologically valid research focusing specifically on free-roaming dog populations.

Future research should prioritize longitudinal and standardized approaches, as well as greater integration between physiological and behavioral methodologies, in order to refine assessment tools and improve comparability across studies.

Overall, advancing integrative and evidence-based approaches to stress assessment and management is essential for improving the welfare of free-roaming dogs and enhancing the effectiveness and sustainability of shelter systems.

## Figures and Tables

**Table 1:** Basal physiological variability of cortisol concentrations in dogs.

Context	Sample Type	Cortisol Pattern	Key Message	Reference
Healthy adult dogs under resting conditions	Blood	Wide inter-individual variability within physiological range	There is no single universal basal cortisol value in dogs	[40]
Clinically healthy dogs prior to procedures	Blood	Baseline levels differ between individuals	Anticipatory factors may influence basal measurements	[41]
Healthy dogs under controlled experimental conditions	Saliva	Stable mean values with individual variation	Salivary cortisol reflects free, biologically active cortisol	[30]
Breed-related comparisons in healthy dogs	Blood	Marked breed-dependent differences	Breed and individual factors significantly affect cortisol levels	[42]
Repeated baseline sampling in healthy dogs	Blood/Saliva	Consistent intra-individual patterns over time	Individual baseline is more informative than population means	[23]

**Table 2:** Cortisol responses in dogs exposed to acute stressors.

Acute Stressor	Sample Type	Cortisol Response	Interpretation	Reference
Transport (road or air)	Blood/Saliva	Rapid increase	Strong activation of the HPA axis during short-term stress	[44]
Veterinary examination	Blood	Transient elevation	Handling and unfamiliar environment act as acute stressors	[23]
Hospitalization and preoperative waiting	Blood	Increased levels prior to procedures	Anticipatory stress response	[41]
Surgical intervention	Blood	Marked perioperative peak	Physiological response to acute clinical stress	[45]
Short-term social isolation	Blood	Temporary increase	Acute disruption of social context triggers stress response	[20]

**Table 3:** Cortisol patterns in dogs exposed to chronic or prolonged stress.

Chronic Stress Context	Sample Type	Cortisol Pattern	Interpretation	Reference
Long-term shelter housing	Blood	Variable or reduced basal levels	Possible HPA axis dysregulation under chronic stress	[20]
Prolonged stay in shelters	Hair	Increased accumulated cortisol	Reflects long-term exposure to stressors	[49]
Chronic social deprivation	Blood/Saliva	Blunted cortisol response	Adaptation or exhaustion of stress response	[37]
Repeated exposure to aversive stimuli	Blood	Inconsistent responses	Chronic stress alters cortisol reactivity	[35]
Shelter dogs with behavioral inhibition	Hair	Elevated long-term cortisol	Association between chronic stress and behavioral changes	[30]

**Table 4:** Behavioral categories of stress in dogs and their welfare implications.

Behavioral Category	Typical Behaviors	Interpretation	Welfare Implications	References
Fear/Anxiety-	Trembling, cowering. Tail tucked	Perception of threat	Acute or chronic stress	[26]
Aggression	Growling, barking, snapping	Defensive coping response	Social conflict or fear	[2]
Withdrawal/Apathy	Hiding, inactivity, lack of interest	Behavioral inhibition	Possible chronic stress	[24]
Stereotypies	Pacing, repetitive movements	Coping with prolonged stress	Reduced welfare	[2]

## Data Availability

No new data were created or analyzed in this study. Data sharing is not applicable to this article.

## Abbreviations

The following abbreviations are used in this manuscript:

HPA	Hypothalamic–Pituitary–Adrenal
HRV	Heart Rate Variability
CRH	Corticotropin-Releasing Hormone
ACTH	Adrenocorticotropic Hormone
